# Supplementary material for: Dynalign II: common secondary structure prediction for RNA homologs with domain insertions
Source: Nucleic Acids Res. 2014 Nov 21;42(22):13939–48. doi: 10.1093/nar/gku1172 (PMC4267632; doi:10.1093/nar/gku1172)
Supplement: SUPPLEMENTARY DATA [file supp_gku1172_nar-02021-z-2014-File012.zip › manual/Text/efn2.html]

RNAstructure Command Line Help: efn2


|  |  |  |
| --- | --- | --- |
|  | RNAstructure Command Line Help  efn2 and efn2-smp | - Contents - Index |
| efn2 (Energy Function 2) is used to calculate the folding free energy change of a structure or structures in a CT file. The output from efn2 can be either a simple list of energies or a detailed breakdown of the calculation. efn2 does not support structures that contain pseudoknots. efn2-smp is a parallel processing version for use on multi-core computers, built using OpenMP. USAGE: efn2 <ct file> <energy file> [options] OR: efn2-smp <ct file> <energy file> [options] Required parameters: <ct file> \| The name of a CT file containing the input structure.; <energy file> \| The energy file to which output is written. The energy file can be written in one of two forms: 1. Simple List Lists free energy for each structure, lowest first. This file type is written by default. 2. Thermodynamic Details Writes details of every substructure in each structure, and corresponding free energy of each. This file type is only written if "-w," "-W," or "--writedetails" (see below) is specified, and replaces a simple list file. Options which don't require added values: -d, -D, --DNA \| Specify that the sequence is DNA, and DNA parameters are to be used. Default is to use RNA parameters.; -h, -H, --help \| Display the usage details message.; -p, -P, --print \| Print the output file to standard output. This won't override default behavior of writing to a file. Thermodynamic files (if written) are not printed, even if this option is specified, because they can be very large.; -w, -W, --writedetails \| Write a thermodynamic details file. The thermodynamic details file replaces a standard output (list) file. Options which require added values: -sh, -SH, --SHAPE \| Specify a SHAPE constraints file to be applied. These constraints specificially use SHAPE pseudoenergy constraints. Default is no SHAPE constraint file specified.; -si, -SI, --SHAPEintercept \| Specify an intercept used with SHAPE constraints. Default is -0.6 kcal/mol.; -sm, -SM, --SHAPEslope \| Specify a slope used with SHAPE constraints. Default is 1.8 kcal/mol.; -t, -T, --temperature \| Specify the temperature at which the free energy free should be calculated in Kelvin. Default is 310.15 K, which is 37 degrees C. Notes for smp: efn2-smp, by default, will use all available compute cores for processing. The number of cores used can be controlled by setting the OMP\_NUM\_THREADS environment variable. References: 1. Reuter, J.S. and Mathews, D.H. "RNAstructure: software for RNA secondary structure prediction and analysis." *BMC Bioinformatics*, 11:129. (2010). 2. Mathews, D.H., Sabina, J., Zuker, M. and Turner, D.H. "Expanded sequence dependence of thermodynamic parameters provides improved prediction of RNA secondary structure." *J. Mol. Biol.*, 288:911-940. (1999). | | |
| Visit The Mathews Lab RNAstructure Page for updates and latest information. | | |
